# Evaluation of Vascular Endothelial Growth Factor Gene Expression in Recellularized Liver Tissue by Mouse Embryo Fibroblast

**DOI:** 10.61186/ibj.3862

**Published:** 2023-06-22

**Authors:** Motahare Homayoon Vala, Hamed Bagheri, Zinat Sargazi, Negar Bakhtiary, Shahram Pourbeiranvand, Mojdeh Salehnia

**Affiliations:** 1 Department of Biomaterials, Faculty of Interdisciplinary Science and Technology, Tarbiat Modares University, Tehran, Iran;; 2 Department of Anatomical Sciences, School of Medical Sciences, Zahedan University, Zahedan, Iran;; 3 Burn Research Center, Iran University of Medical Sciences, Tehran 1449614535, Iran;; 4 Department of Anatomical Sciences, School of Medical Sciences, Tarbiat Modares University, Tehran, Iran

**Keywords:** Vascular endothelial growth factor, Gene expression, Fibroblast, Liver, Decellularized ECM

## Abstract

**Background:**

The aim of the present study was to evaluate alterations in the *vegf* gene expression as an angiogenic factor in mouse embryo fibroblasts seeded on the decellularized liver fragments.

**Methods:**

Liver tissue samples (n = 10) collected from adult male mice were randomly divided into decellularized and native control groups. Tissues were decellularized by treating with 1% Triton X-100 and 0.1% SDS for 24 hours and assessed by H&E staining and SEM. Then DNA content analysis and toxicity tests were performed. By centrifugation, DiI-labeled mouse embryo fibroblasts were seeded on each scaffold and cultured for one week. The recellularized scaffolds were studied by H&E staining, SEM, and LSCM. After RNA extraction and cDNA synthesis, the expression of the *vegf *gene in these samples was investigated using real-time RT-PCR.

**Results:**

Our observations showed that the decellularized tissues had morphology and porous structure similar to the control group, and their DNA content significantly reduced (p < 0.05) and reached to 4.12% of the control group. The MTT test indicated no significant cellular toxicity for the decellularized scaffolds. Light microscopy, SEM, and LSCM observations confirmed the attachment and penetration of embryonic fibroblast cells on the surface and into different depths of the scaffolds. There was no statistically significant difference in terms of *vegf *gene expression in the cultured cells in the presence and absence of a scaffold.

**Conclusion:**

The reconstructed scaffold had no effect on *vegf* gene expression. Decellularized mouse liver tissue recellularized by embryonic fibroblasts could have an application in regenerative medicine.

## INTRODUCTION

Decellularized tissue as an alternative scaffold for repairing damaged tissues has received special attention in tissue engineering and regenerative medicine^[^^1^^-^^3^^]^. Owing to the high incidence of liver diseases and limitations in the preparation of liver tissues, DLS has recently been recognized as an effective substrate for transplantation of engineered liver tissue^[^^4^^-^^7^^]^. Decellularized scaffolds can be manufactured through eliminating cellular components from the native tissue using detergent perfusion and enzyme solutions and/or physical methods^[^^1^^,^^2^^]^. Triton X-100 and SDS are common detergents that are widely used by investigators for the removal of cellular materials^[^^8^^-^^11^^]^. While the cells are destroyed by these detergents, the structure of decellularized liver tissue is well preserved. In DLS, critical molecules that involve in cell attachment, migration, and proliferation, such as glycoproteins, proteoglycans, and polysaccharides, are maintained^[^^12^^]^. Our recent study has demonstrated that the application of Triton X-100 in combination with 0.1% SDS for 24 hours for decellularization of mouse liver fragments, results in favorable preservation of tissue morphology and the ECM molecules similar to the native control^[^^11^^]^. 

Various applications have been introduced for DLSs according to their characteristics. These scaffolds could provide an appropriate substrate for cell support and attachment during in vitro cell culture^[^^13^^,^^14^^]^. Recently, combination of decellularized liver tissue with other types of natural or synthetic scaffolds in a three-dimensional matrix has been utilized for supporting the cell culture, which improves the cell viability and function^[^^14^^,^^15^^]^.

Considering the high ability to adhere the cells, liver scaffolds are applied for the treatment of hepatobiliary diseases and liver cancer^[^^16^^]^. In recent years, improvement of two- and three-dimensional culture systems in laboratory conditions, as well as the use of organ-on-a-chip technology has led to advancements in the treatment of liver-related diseases^[^^10^^,^^17^^,^^18^^]^. The liver scaffolds provide a suitable reservoir of signaling molecules, cytokines, and growth factors, which are critical and essential for the cellular growth, proliferation, and differentiation^[^^19^^]^. For the mentioned purposes, the decellularized liver tissues are repopulated by several methods and using a variety of somatic and stem cells^[^^10^^,^^13^^-^^15^^,^^18^^,^^19^^]^. The main sources of cells applied for repopulating the decellularized liver tissue are primary hepatocytes derived from embryonic or adult tissues and mesenchymal stem cells obtained from bone marrow or adipose tissues^[^^10^^,^^11^^-^^15^^,^^18^^,^^19^^]^. Our previous study demonstrated that the mouse DLS facilitated the adhesion and penetration of human endometrial mesenchymal cells^[^^11^^]^. Angiogenesis plays a crucial role in regeneration of damaged tissues, women's menstruation, pregnancy, and wound healing, and insufficient angiogenesis results in impaired healing of damaged tissues^[^^20^^]^. Angiogenesis in both the embryonic period and adulthood has an important role in transferring growth and survival factors. The interaction of angiogenesis-related factors with ECM molecules regulates the expansion of the capillary network^[^^20^^-^^23^^]^. Some growth factors and mediators are involved in differentiation and proliferation of endothelial cells, including VEGF^[^^24^^]^. As an angiogenic factor, VEGF has mitogenic and antiapoptotic effects on the endothelial cells, as well as increases vascular permeability and contributes to the proliferation, survival, and migration of cells in the physiological processes such as pregnancy, wound healing, and menstruation after birth^[^^24^^-^^26^^]^.

The MEF is derived from embryonic mesenchymal cells and is capable of differentiating into several cell types, including stromal and endothelial cells; it also has a critical role in wound healing^[^^19^^]^. Due to easy access and rapid growth rate, MEF cell culture is an excellent tool for a wide range of experiments^[^^27^^-^^29^^]^. The recellularized liver scaffolds could act as a cell delivery carrier, especially once involves in angiogenesis to improve damaged tissues^[^^6^^,^^10^^,^^13^^-^^15^^,^^30^^-^^32^^]^. Based on the critical function of the cell microenvironment (niche) in the regulation of cell behavior, the present study, for the first time, evaluated attachment and penetration of MEF to the decellularized scaffold. In addition, the inductive effect of this scaffold on the *vegf* gene expression was analyzed in the cells seeded after one week of cultivation.

## MATERIALS AND METHODS

All materials and reagents were obtained from Sigma Aldrich (London, UK), otherwise they were mentioned within the text.

**Collection of liver samples**

The liver tissues obtained from 10 adult male NMRI mice by cervical dislocation were washed thoroughly in PBS and dissected into 1 × 1 × 3 mm^3^ fragments. 

**Decellularization and recellularization of liver fragments**

Decellularization of mouse liver tissue fragments (n = 12) was achieved using a combination of Triton X-100 and 1% SDS as described previously^[^^11^^]^. Briefly, fragments of liver tissue were placed in Triton X-100 on a stirrer at 50 rpm for 24 hours, after washing with PBS. The fragments were then placed in 0.1% SDS in a shaker at 50 rpm for 24 hours. Following the decellularization process, all the samples were washed in PBS for 24 hours and assessed by the light microscopy and DNA content analysis. Recellularization was carried out by in vitro culture of the MEF cells for one week. The cell attachment and penetration to into this scaffold were then studied by H&E staining, SEM, and LSCM. Finally, *vegf* gene expression was evaluated by using real-time RT-PCR, and the scaffold ultrastructure and cytotoxicity were assessed by SEM and MTT test, respectively. 

**Light microscopic observation by H&E** **staining**

Decellularized tissue fragments (n = 3) were fixed in 10% neutral-buffered formalin, dehydrated in increasing concentrations of alcohols (70, 80, 90, and 100%) and embedded in paraffin wax after being kept in xylene for 1 hour. After cutting the tissues at a thickness of 4 µm, H&E staining was applied.

**DNA content analysis**

To extract DNA content, we digested the samples (n = 3) from the decellularized and the control groups with TRIzol® reagent (Invitrogen; n = 3 for each group in three repeats). The solution was then centrifuged at 2000 ×g at 4°C for 10 minutes, and the supernatant was removed. Afterwards, the chloroform was added, and the supernatants were centrifuged again for 10 minutes. In the next step, the middle part of the solution was collected and the absolute alcohol was added. Subsequently, sodium citrate was added to the samples, followed by the addition of 75% ethanol. Finally, the samples were resuspended in NaOH. After centrifugation for 10 minutes, DNA content was determined (ng/mg wet tissue) by UV spectro-photometry (Eppendorf, Germany) at 260 nm of optical density^[^^11^^]^. 

**MEF preparation**

The MEF cells were obtained from the Cryobank of the Anatomy Department of Tarbiat Modares University, Tehran, Iran. The cells were cultured in DMEM medium containing 10% fetal bovine serum (Gibco, UK), 100 of IU/ml penicillin, and 75 of µg/ml streptomycin and kept at 5% CO_2_ at 37 °C for 48 hours until confluency. The MEF cells were then trypsinized and used for the MTT assay and cell seeding.

**MTT assay**

The cytotoxicity of the decellularized scaffolds was assessed using the MTT test according to the MTT cell proliferation and cytotoxicity assay Kit (Abcam, Jermany; Ab211091). The decellularized scaffold was sterilized by keeping it in 70% ethanol for 30 minutes, followed by three times washings with PBS containing 100 IU of penicillin and 75 µg/ml of streptomycin. A total of 2 × 10^4^ MEF cells were cultured in 96-well plates, in the presence and absence of the decellularized scaffold. After 48 and 72 hours, the scaffolds were removed, and the remaining cells were incubated with MTT solution for 4 hours. The optical density was measured at 570 nm using a microplate reader (Biochrom, Germany). These experiments were performed in three repeats in each time points (48 and 72 hours) for both studied groups. 

**SEM study**

To investigate the ultrastructure of decellularized and normal tissues, three samples were collected from each group and fixed in 2.5% glutaraldehyde for 2 hours. Next, the samples were dehydrated by ethanol and hexamethyldisilazane (Merck, Germany), respectively. Finally, the samples were coated with gold and observed under a scanning electron microscpoe (VEGA/ TESCAN-XMU, Czech Republic).

**Recellularization of scaffolds**

For recellularization and sterilization of the scaffolds, MEF cells were seeded on 12 scaffolds (3 × 1 × 1 mm^3^) using a centrifugal method at 2500 ×g at 37 °C for 5 minutes. The cells were then cultured in DMEM-F12 medium supplemented with 10% fetal bovine serum, 100 IU/ml of penicillin, and 75 µg/ml of streptomycin and incubated in 5% CO_2_ at 37 °C and for 1 week. 

**Light microscopy, SEM, and** **LSCM** **studies of recellularized scaffold**

At the end of cultivation time, three pieces of the scaffolds were fixed in 10% neutral buffered formalin, processed for H&E staining and visualized under light microscopy. For SEM study, the recellularized scaffolds (n = 3 samples) were fixed and processed as described earlier in the Section “SEM study”. In another series of experiments, the MEF cells were labeled with DiI fluorescent dye. Briefly, 1 × 10^5^ cells/ml were maintained in Dil (2 μg/ml; Invitrogen) at room temperature and then placed in a dark place at 4 °C for 20 min. Then the cells were washed three times in PBS and seeded on the scaffold. After one-week of in vitro culture, the cells were examined by LSCM (ZEISS LSCM 800, Germany) with excitation wavelength of 546 nm and emission wavelength of 563 nm. 

**Real-time RT-PCR**

The gene expression of *vegf* was assessed using real-time RT-PCR. Designing primers for *vefg *as target gene and *β-actin* as housekeeping gene were carried out by using Allel ID 7.8 software (Table 1). According to the manufacturer's protocol (Sinaclon, IRAN), total RNA was extracted from MEF cells growing on the scaffolds (n = 3) as experimental group, and the cultured MEF cells in the absence of scaffold were used as a control group (n = 3). The quantity of RNA was evaluated by spectrophotometry, and cDNA was synthesized using oligo(dT) primers and a cDNA synthesis kit (Thermo Scientific, USA). Then the relative mRNA level expression of target genes to control gene was determined using QuantiTect SYBR Green RT-PCR Kit (Amplicon, Denmark) and calculated using the 2^-ΔΔCT^ method.

**Table 1:** Primer sequences used for real-time RT-PCR

**Target gene**	**Primer sequence**	**PCR product size (bp)**
*VEGF*	F: CTGCACCCACGACAGAAGG R: AGCTTCGCTGGTAGACATCC	72
*Β-actin*	F: AGTCATAGTCCGCCTAGAAGC R: TGAAGATCAAGATCATTGCTCCC	68

**Statistical analysis**

Statistical analyses were carried out by using SPSS software (V24; SPSS Inc., Chicago, USA). Data of DNA content, MTT assay, and molecular analysis were compared using t-tests and one-way analysis of variance (ANOVA), followed by post hoc Tukey's test. The significance level was statistically set at p ≤ 0.05. Each experiment was conducted at least in triplicate.

## RESULTS

**Morphology of decellularized samples**

**Fig. 1:**
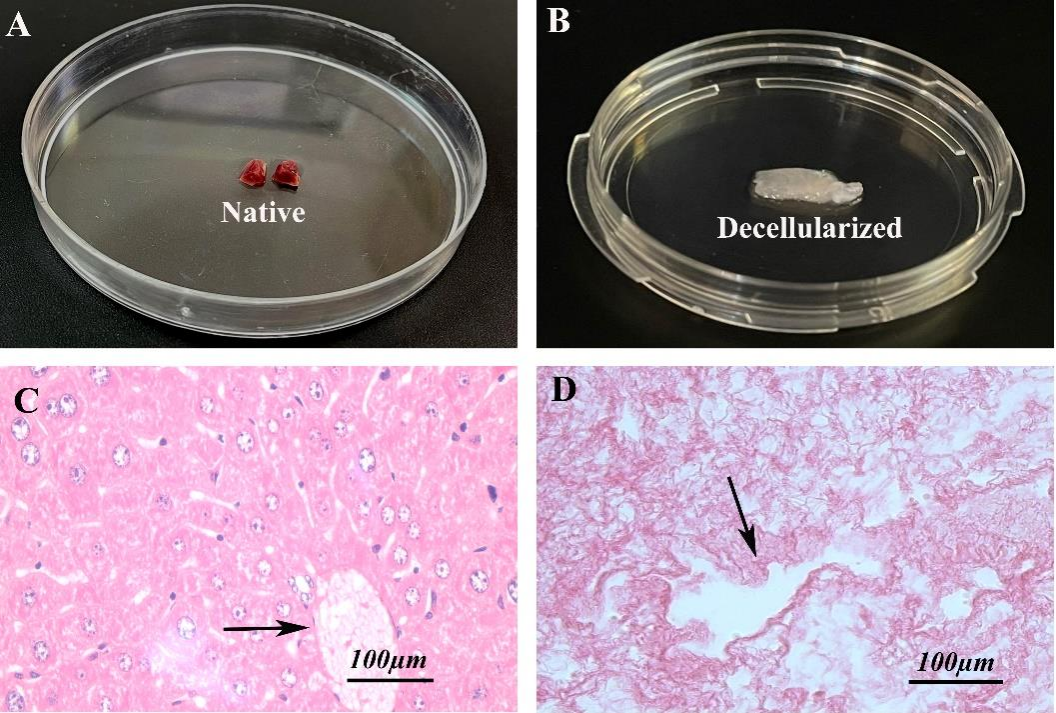
Representative images of liver tissue. The macroscopic and ligh microscopic (using H&E staining) observations of native control (A and C) and decellularized liver tissue (B and D). The black arrows indicate the central vein

As the Figure 1A shows, the macroscopic appearance of native intact control group shows pinkish color, while that of the decellularized mouse liver samples was white (colorless) and transparent (Fig. 1B). In spite of the presence of normal hepatocytes and endothelial cells within the native control tissue, there was no nucleus in the decellularized tissue sections (Fig. 1C and 1D). The hepatocytes in the control group arranged in rows toward the central vein. In addition, the tissue structure and collagen fibers were well preserved throughout the decellularized samples similar to the control. 

**Fig. 2:**
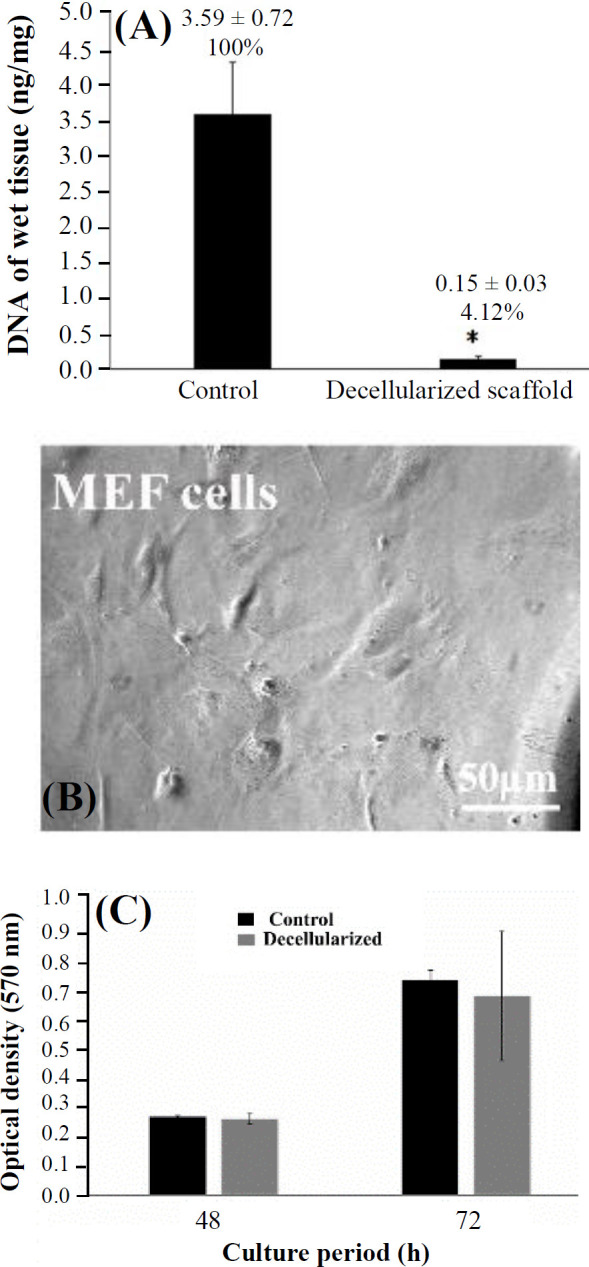
(A) comparison of DNA content between the control and the decellularized groups (^*^p ≤ 0.05 vs. the control group); (B) MEF morphology under an inverted microscope; (C) the results of MTT assay in the control and decellularized groups after 48 and 72 hours. There was no significant difference between the two groups

**Comparison of DNA content between groups**

As depicted in the Figure 2A, the amounts of DNA in the control and decellularized groups were 3.59 ± 0.72 ng/mg (100%) and 0.15 ± 0.03 ng/mg (4.12%) of wet tissue, respectively. This amount significantly decreased by 95.82% in the decellularized sample compared to the control group (p ≤ 0.05).

**Inverted microscope observation of MEF cells**

After 24 hours of in vitro culture, the MEF cells adhered to the floor of the flask, showed a spindle-shaped appearance (Fig. 2B). 

**MTT assay results**

The optical density of the samples after 24 and 72 hours of culturing were 0.26 ± 0.004 and 0.74 ± 0.03 in the control group and 0.26 ± 0.02 and 0.68 ± 0.23 in the decellularized group, respectively. There were no significant differences among these groups (Fig. 2C).

**SEM observation**

**Fig. 3:**
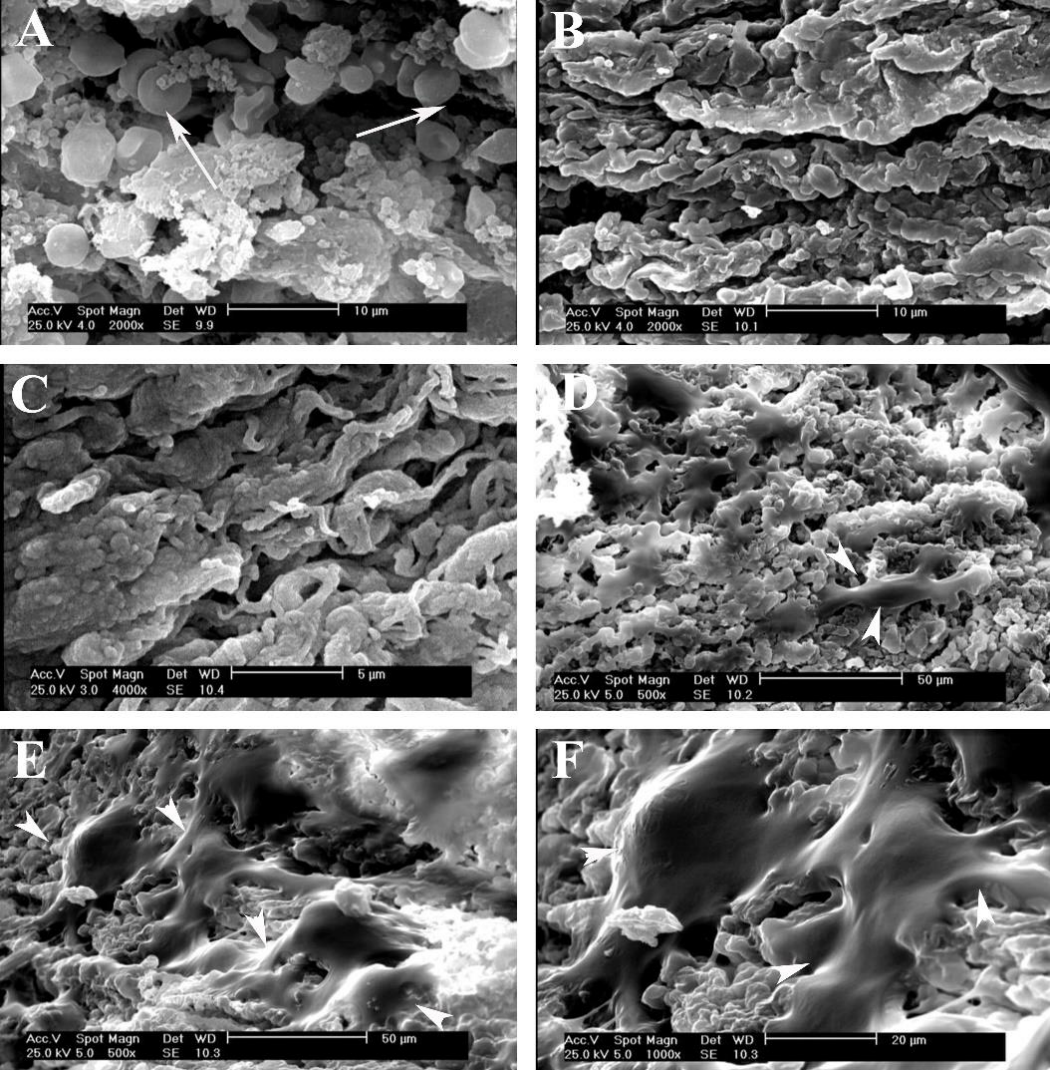
Representative micrographs of the studied group by SEM. (A) In the native control liver tissue, the red blood cells are indicated by the white arrows; (B and C) the ultrastructure of the DLS before cell seeding; (D-F) the recellularized liver scaffolds by MEF cells, with low- and high-power magnification. White arrowheads show the attached cells

Electromicrographs of control and decellularized mouse liver tissue with low- and high-power magnifications are presented in Figure 3A-3C. The presence of blood cells within the sinosuoid of native control tissue are obvious (Fig. 3A), while there is no evidence of the cells within the decellularized group. The collagen fibers and poros structure had similar appearance in the decellularized and normal tissues. The SEM images of recellularized scaffolds after cell seeding with MEF confirmed the presence of these cells. As shown in Figure 3D-3F, the cells were attached and spread on different areas of the scaffold. The attached cells have several long processes that are extended on the surface of scaffold (Fig. 3E and 3F). 

**Light microscopic observation of recellularized liver scaffolds**

**Fig. 4:**
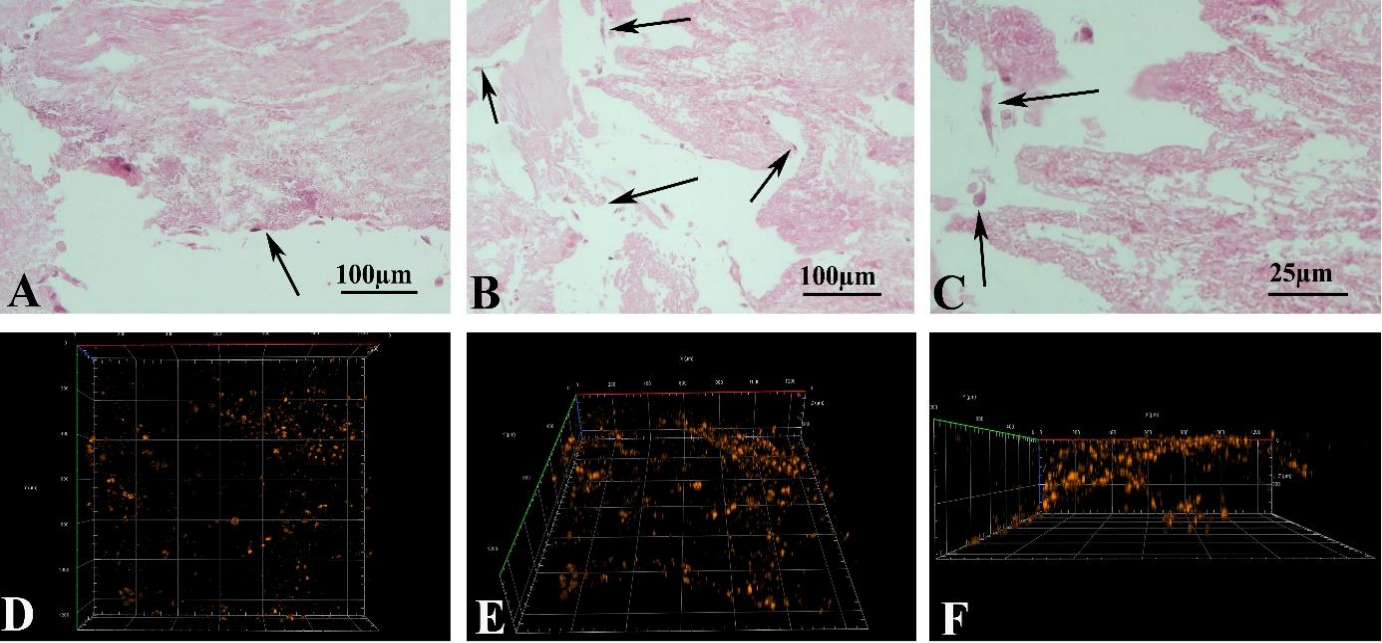
(A-C) The morphology of recellularized scaffolds after H&E staining, showing that the cells were attached and penetrated within the scaffold (black arrows); (D-F) Three-dimensional micrographs captured by using a laser scanning confocal microscope. They (These figures show the presence of Dil-labeled MEF cells within the scaffold, and they are visible at 200 micrometer depth.) confirmed the presence of DiI-labeled cells within the scaffold, and the cells are visible at a depth of 200 μm

As shown in Figure 4A-4C, the cells were observed at the periphery of scaffold. Some of the penetrated cells were also observed within different parts of the recellularized scaffold after one-week of cultivation. 

**Observation of scaffold using LSCM**

Two- and three-dimensional micrographs of recellularized tissue under the LSCM are illustrated in Figure 4D-4F. The DiI labeled cells are red in color. The cells were penetrated and scattered into the scaffold to 200 μm depth.

**Real-time RT-PCR results**

**Fig. 5:**
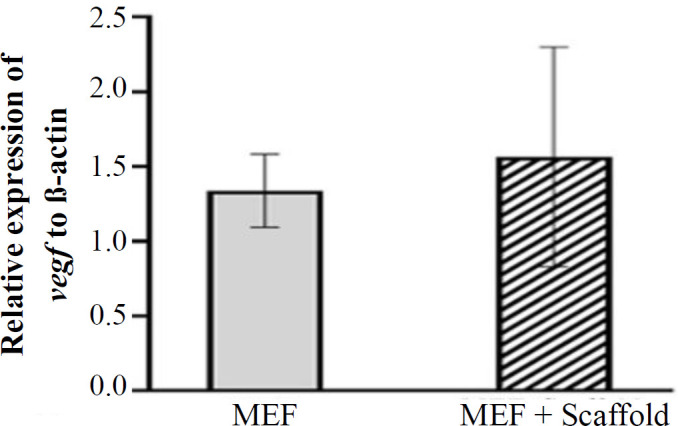
Comparison of the expression ratio of *vegf *gene in recellularized scaffolds in the MEF and control groups. There was no significant difference between the two groups

The expression ratio of *vegf* to *β-actin* genes in the seeded MEF cells on the scaffolds and the control group are illustrated in Figure 5. As depicted in this Figure, the relative expression of *vegf* gene in the recellularized and control groups was 1.57 ± 0.72 and 1.33 ± 0.24, respectively. There was no significant difference between these two groups (p ≤ 0.05). 

## DISCUSSION

Our morphological and SEM observations of the decellularized tissue revealed that collagen fibers and stroma were well preserved and the porosity were similar to the control group. In agreement with a previous research in this field, the applied decellularization method in the present study by using Triton X-100 and SDS was found to be effective for preserving tissue structure and the removal of cellular component^[^^33^^]^. Triton X-100 has polar head that disrupts hydrogen bonds of lipids, but it is uncapable to remove the protein-protein bond; therefore, SDS treatment was applied for complete removal of the native cells^[^^34^^,^^35^^]^. In other part of this study, the analysis of DNA content demonstrated a significant reduction in DNA content of the created scaffolds, which is comparable to other investigations^[^^1^^,^^4^^-^^8^^]^. Moreover, the MTT assay results revealed that the decellularized liver scaffolds were non-toxic and had no negative effect on the cell survival. 

A variety of somatic and stem cell sources have utilized for re-cellularization of liver scaffolds^[^^10^^,^^13^^-^^18^^,^^30^^-^^32^^]^. For the first time in the present study, we seeded the MEF cells on the decellularized liver tissue, and our observations indicated that these cells were adhered on the surface and penetrated into the scaffold. These results suggested that the ECM components involved in the cell attachment and migration were well preserved in the created decellularized tissue. In agreement with this observation, analysis by Raman spectroscopy showed that the amount of collagen type I and IV did not reduce in the decellularized samples^[^^11^^]^. 

The micrographs captured by SEM and LSCM revealed that the MEFS cells were adhered and penetrated properly into the liver scaffold. The liver niche has potentially unique properties such as providing suitable substrates for cell attachment, growth, and proliferation, and it has the ability to induce angiogenesis^[^^5^^,^^36^^,^^37^^]^. The adhesion of the cell to matrix is mediated by several type of receptors, including integrins molecules that interact with their ligands such as fibronectin, laminin, and collagen^[^^37^^]^. 

Some similarities were observed between MEFS and mesenchymal stem cells, such as their differentiation into different cell types^[^^27^^,^^38^^,^^39^^]^. At the molecular level, we evaluated the differentiation of MEF cells into angioblastic cells by analyzing the alteration in the expression of *vegf* gene in the seeded cells after one week of culture. However, our molecular results demonstrated that the DLS did not have any inductive effect on increase of the *vegf* expression in the cultured cells. One explanation for these results is that during decellularization of liver tissue, the angiogenic inducer factors may be destroyed; thus, they had no influence on the expression of *vegf* gene in the seeded cells. In spite of this result, it is suggested that DLS can be used as a suitable carrier for releasing the MEF cells in damage tissue. Similarly, Alexanian et al.^[^^38^^]^, by seeding the mouse fibroblast on decellularized mouse hearts and lungs for a period of 10-12 weeks, indicated that these cells are capable to differentiate into myocardiocytes. Therefore, it seems that MEF cells could contribute to wound healing and repair damage tissue by differentiating into other necessary cells. Our previous research has also demonstrated that the human endometrial mesenchymal cells can successfully recellularize the liver scaffold, and this reconstructed structure has potentially clinical applications in the future^[^^11^^]^.

In conclusion, the DLSs provide proper cell homing for MEF with potential application in regenerative medicine. The reconstructed scaffold also has no effect on the *vegf* gene expression.

## DECLARATIONS

### Acknowledgments

This research project was extracted from a M.Sc. thesis.

### Ethical statement

The study protocol was approved by the Ethics Committee of Tarbiat Modares University, Tehran, Iran (ethical code: IR.MODARES.REC.1400.186). 

### Data availability

The raw data supporting the conclusions of this article are available from the corresponding author upon reasonable request. 

### Author contributions

MHV: performed the experiments; HB: co-supervised and involved in protocol development; ZS: involved in preparation of samples for LSCM and LM; NB: involved in writing the original draft; SP: involved in molecular experiments; MS: supervised the study and performed the writing of the manuscript. All authors reviewed and edited the manuscript and approved the final version of the manuscript.

### Conflict of interest

The authors declare that the research was conducted in the absence of any commercial or financial relationships that could be construed as a potential conflict of interest.

### Funding/support

This study was founded and supported by Tarbiat Modares University, Tehran, Iran.
